# Computer Guided Implantology Accuracy and Complications

**DOI:** 10.1155/2013/701421

**Published:** 2013-09-03

**Authors:** Vincenzo Bruno, Mauro Badino, Francesco Riccitiello, Gianrico Spagnuolo, Massimo Amato

**Affiliations:** ^1^Department of Neurosciences, Reproductive and Odontostomatological Sciences, University of Naples Federico II, Naples, Italy; ^2^Medical School, University of Salerno, Salerno, Italy

## Abstract

The computer-based method allows the computerized planning of a surgical implantology procedure, using computed tomography (CT) of the maxillary bones and prosthesis. This procedure, however, is not error-free, unless the operator has been well trained and strictly follows the protocol. A 70-year-old woman whom was edentulous asked for a lower jaw implant-supported prosthesis. A computer-guided surgery was planned with an immediate loading according to the NobelGuide technique. However, prior to surgery, new dentures were constructed to adjust the vertical dimension. An interim screwed metal-resin prosthesis was delivered just after the surgery; however, after only two weeks, it was removed because of a complication. Finally, a screwed implant bridge was delivered. The computer guided surgery is a useful procedure when based on an accurate 3D CT-based image data and an implant planning software which minimizes errors.

## 1. Introduction

The computer-based technique allows the virtual planning of a surgical implantology procedure by using a CT/CBCT of the maxillary bones and prosthesis [1–3] with an analysis of the jaw in all three dimensions. Therefore, it is possible to plan a precise position of the implant respect to the anatomical structures as well as the prostheses, improving the predictability of implant placement, obtaining a prosthetically driven planning [4]. An accurate surgical guide is generated that is also used by the dental technician to fabricate a definitive cast with the precise position of the implants before the surgery. Therefore, a definitive or interim prosthesis can be constructed prior to the surgical procedure, therefore, obtaining an immediate function. 

The NobelGuide (Nobel Biocare AB, Göteborg, Sweden) technique permits the surgical installation of the implants without raising a flap, reducing significantly the operating time and the impact on the patient and patient's morbidity (postoperative pain and swelling) [5]. Several studies [3, 6, 7] reported a cumulative survival rate similar to the conventional surgical procedures, with a few complications.

In the present case report, immediate implant borne prostheses were construct via a computer-assisted implantology and prosthodontics; however, a late surgical complication occurred and in turn CAD/CAM titanium implant bridge was delivered.

## 2. Case Presentation and Results

**Figure 1:**
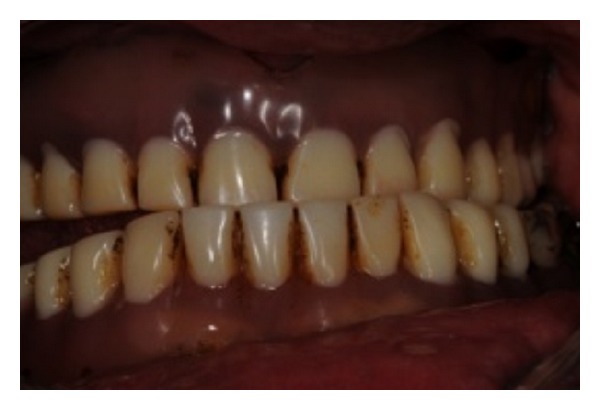
Initial dentures worn out with an altered occlusion.

**Figure 2:**
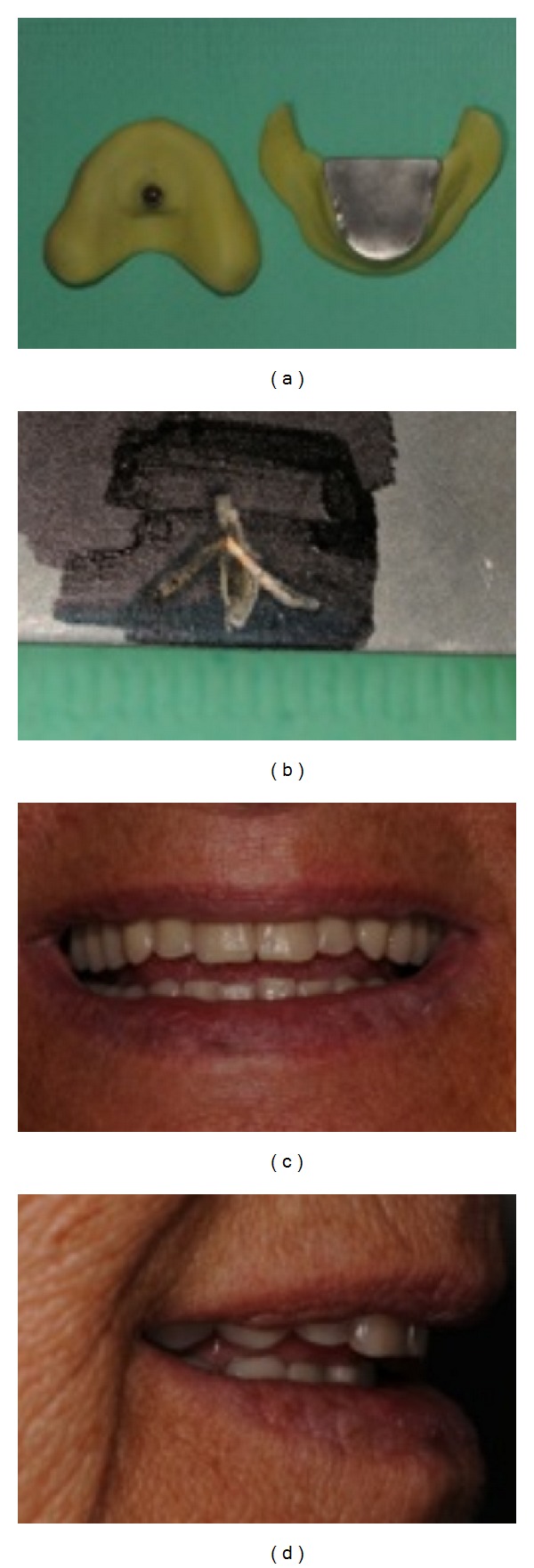
Registration of the gothic arch, according to central bearing point (CBP), to restore the correct interarch relationships ((a), (b)), the new dentures in use ((c), (d)).

A 70-year-old Caucasian woman came into our practice and was in good physical health with no medical risks. She had been wearing two complete dentures (Figure 1) and desired to rehabilitate her edentulous jaws by an implant-retained prosthesis. Two new complete dentures were manufactured to adjust the vertical and horizontal dimensions and to enhance her aesthetic outlook (Figures 2(a), 2(b), 2(c), and 2(d)).

**Figure 3:**
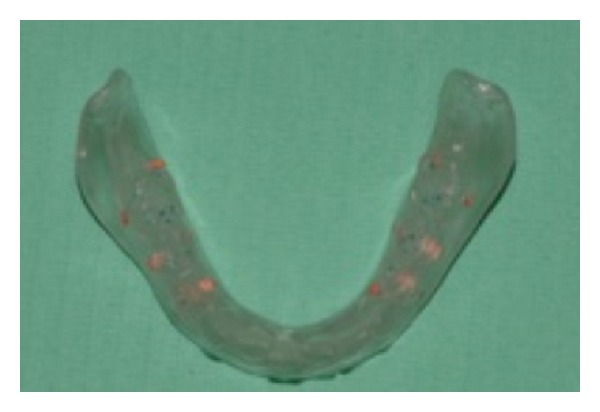
The low denture duplicated and with the gutta-percha markers to be used for the CT.

**Figure 4:**
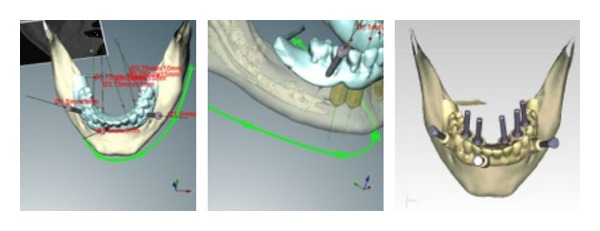
Representative images from the software with the driven prosthetic implants planned.

A panoramic radiograph and two spiral computed tomograms (CT) were taken. The first CT was taken of the mandible with the prostheses and a silicon index for the occlusion. The second CT was taken of the denture duplication, using gutta-percha markers (Figure 3). The double scanning system is necessary, because the density of the radiographic mask is similar to that of soft tissues, and a single scan might not be sufficient to distinguish between the two. After loading the two tomograms into the NobelGuide software (Nobel Biocare, AB), the images were converted and coupled. Then, three-dimensional (3D) planning was performed, which entails positioning the implants in accordance with anatomical structures, such as the mental foramina and mandibular nerve and the prostheses (prosthetic driven planning) (Figure 4).

The surgical template and laboratory products were ordered, allowing the dental technician to produce a definitive cast model with an accurate position for future implants, obtained from the surgical template. Therefore, this model reproduces the jaw bone and dental prosthesis with a space in between representing the gingiva and mucosa. The cast was then mounted on an articulator using the prostheses replica and the bite index. A reinforced interim prosthesis was made through the diagnostic waxing obtained from the previous prostheses. 

**Figure 5:**
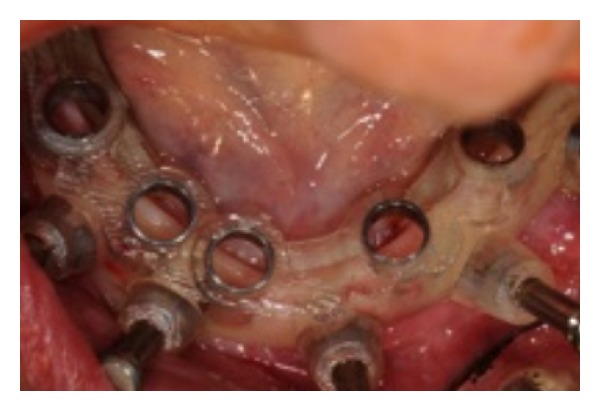
The surgical template in situ.

On the day of surgery, preoperative antibiotics (Amoxicillin 1 g 12 hourly) were given orally 1 day prior to surgery and were continued for another 5 days postoperatively. Local anesthetic was administered, and the surgical template was blocked intraorally using pins (Figure 5), according the position obtained from the casts mounted in articulator. In particular, a surgical bite, to transfer the position, is fabricated from the casts previously mounted in articulator. The gingiva was cut using a circular scalpel, and the osteotomies were performed by following strict protocol [2]. According to the protocol, the osteotomes for the implants were prepared through metallic cylinders which are inserted in the template using a series of drilling sleeves with different diameters that completely coincide with the series of twist drills, taps, and implant holders/mounts. Thus, the drilling is guided and the drill can be moved only along the planned axis avoiding any wobbling. Using this protocol the implant position is precisely transferred from digital position to the mouth.

**Figure 6:**
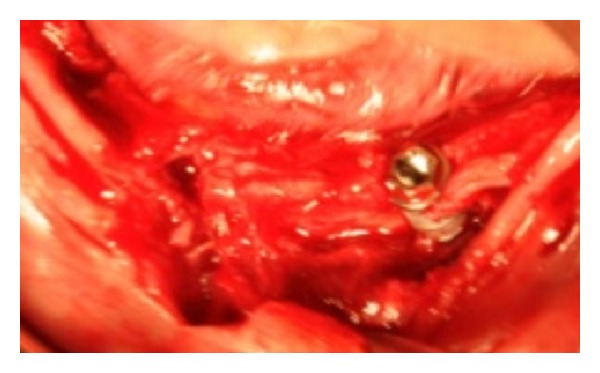
An image, out of focus, with the implants' position too buccal.

**Figure 7:**
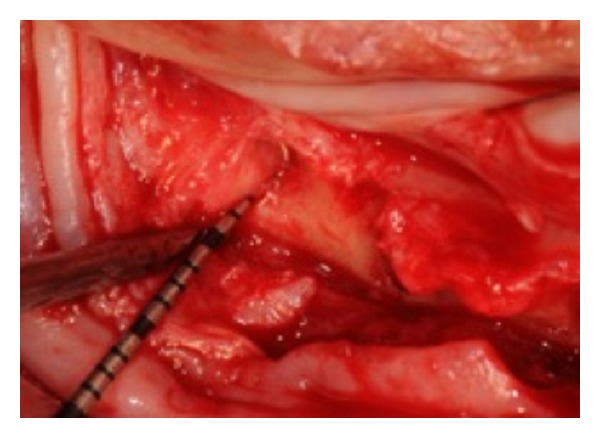
The distal osteotome intimate to the alveolar nerve.

**Figure 8:**
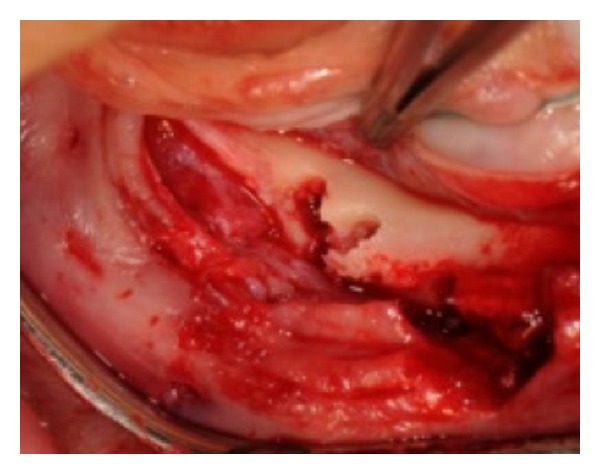
The two osteotomes very close.

**Figure 9:**
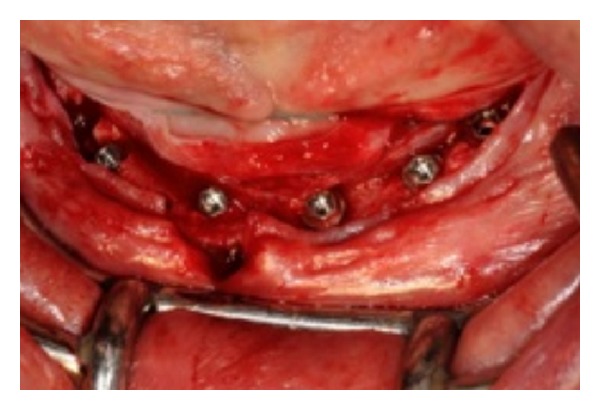
All the five implants placed free hand.

**Figure 10:**
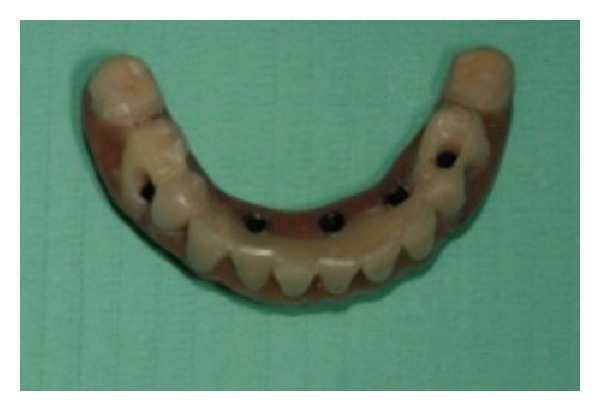
The denture modified to be used as interim implant borne prosthesis.

The first two implants were positioned, but good primary stability was not established. The template was removed, showing that the implants were positioned buccally (Figure 6). The implants were then unscrewed/removed, and the operator made the decision to continue the surgery free hand and open flap. A new osteotomy was performed distally, but this action resulted in being too close to the mandibular foramina (Figure 7); therefore another osteotomy was performed mesially (Figure 8). The other osteotomies were performed, and five implants were placed (Figure 9). Finally, the last denture was adapted to the new implant positions (Figure 10), and the reinforced interim prostheses fabricated could not be delivered. 

**Figure 11:**
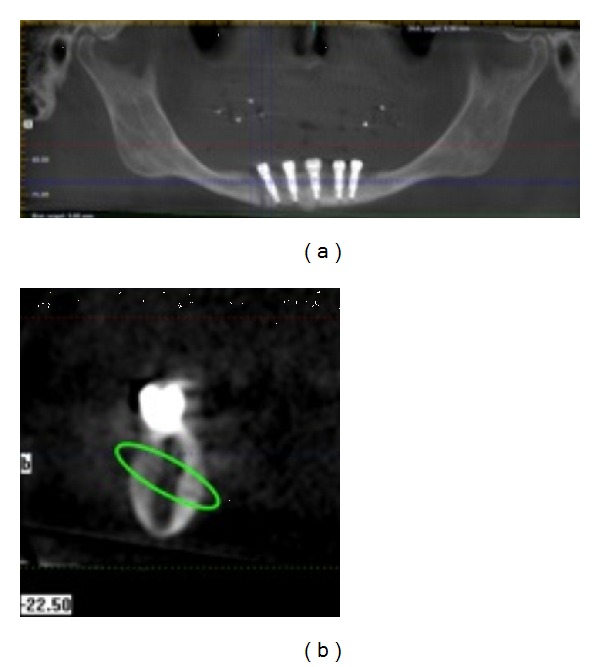
The CBCT (a) and a slice (b) where the hairline fracture is evident.

**Figure 12:**
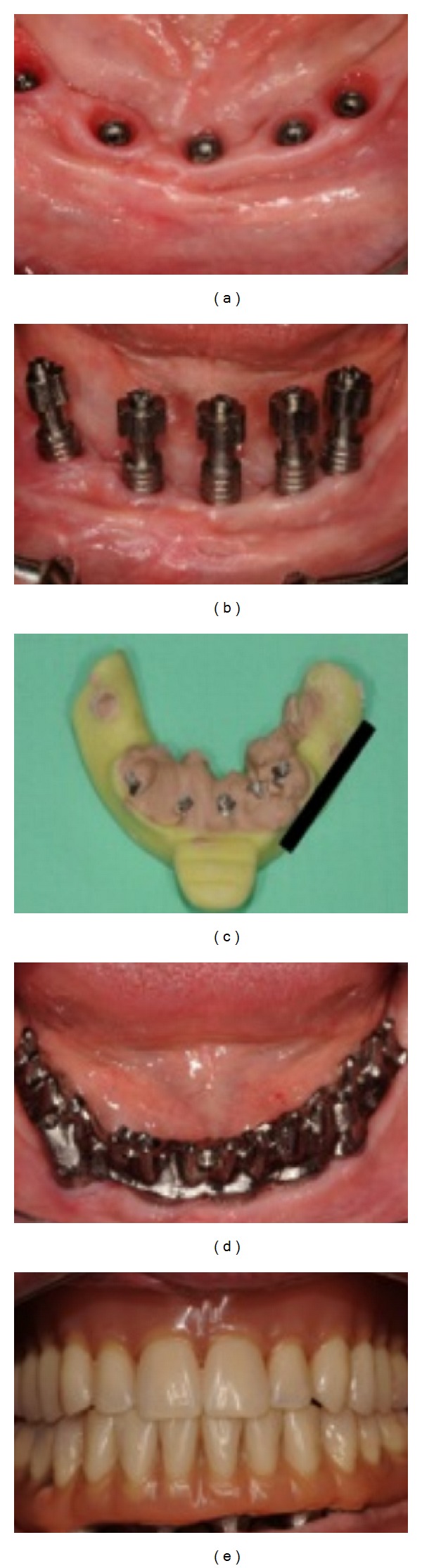
The healed mucosa and the osseointegrated implants (a). The impression abutments where the angulation of the distal right implant is inserted and evident (b). The impression was taken using plaster (c). The try-in of the screwed titanium implant bridge (d). The titanium implant bridge delivered (e).

Two weeks later, the patient called stating that while she made a wide yawn, she heard a noise in the mandible and currently has pain with any movement. A Cone Beam CT was taken, and a hairline fracture was detected (Figures 11(a) and 11(b)). The patient was then referred to a maxillofacial surgeon, who stated that no surgery was required. However, the prostheses needed to be unscrewed, to allow the mandible to rest, and the patient was asked to follow a soft diet for 2 months. Two months later, a CAD/CAM titanium implant bridge was constructed to complete the procedure (Figures 12(a), 12(b), 12(c), 12(d), and 12(e)).

## 3. Discussion

Van Steenberghe et al., 2005 [2], by using an immediately loaded CAD-CAM manufactured fixed complete dentures computer-designed procedure, reported an implant cumulative survival rate after 5 years of 91.5%. In particular, there was a difference between the nonsmoking group (98.9%) and the smoking group (81.2%), with a mean marginal bone loss of 2.6 mm for the smoking group and 1.2 mm for the nonsmoking group. 

Gillot et al., 2010 [7], reported an implant survival rate of 98.1%, and of the 211 implants loaded, four were lost (1.9%). Even so, the prosthetic survival rate was 100%. It is still interesting to note that the most commonly used implant lengths were between 10 and 13 mm; these lengths represent 75% of the overall pool of implants, and in particular, the 13 mm and 15 mm lengths represent the 52.13% [7]. Therefore, a conclusion can be drawn that good results can be obtained with the use of longer implants. 

Vasak et al., 2012 [8], presented similar results regarding the implant survival rate (98.8%), adding more details concerning the mean marginal bone level at 1 year (1.44 mm; SD 1.35; *n* = 98) and reporting an increment of all the clinical parameters of the soft tissue conditions.

The flapless surgery, based on keyhole surgery, entails less intense pain and for shorter periods of time [5]. Moreover, this minimal invasive technique reduces surgery time and swelling, compared to conventional treatment [5]. This treatment also reduces the number of appointments and chair time for the patient as well as for the dentist.

However, prosthetic and surgical complications [9] are reported in the literature using the computer-aided implantology. Authors [9] have also stated that it is particularly important to have a “strict adherence” to the protocol, to prevent any complications. Schneider et al. [10] reported early surgical complications such as the lack of primary stability, which has been resolved by the use of wider implants, bone augmentation, or no loading. 

D'haese et al. [11], in a literature review, reported that there is a substantial deviation between the implant planned and the one placed. This deviation can vary from a mean angular of 2.71° (range 0.4–8, SD 1.9), with a mean deviation at the apex of 1.0 mm (range 0.2–3.0, SD 0.7) [11], to 7.9° (range 0.7–24.9, SD 4.7), with a mean deviation at the apex of 1.6 mm (range 0–4.2, SD 1.2) [12].

Another point to consider is the deformation of the stereolithographic surgical guide caused by the incorrect setting of the ISO values for the segmentation of the scan denture [13]. Stumpel concluded that the evaluation of the produced surgical guide before the clinical use might be prudent [13]. To avoid this arbitrary setting, Nobel Biocare has introduced the calibration procedure using a unique calibration object. This procedure permits obtaining a calibration of the scan in relation of the object, resulting in a production of surgical templates with a better stability and precision.

We would also like to point out that in this case the protocol was strictly followed and the surgeon was extremely experienced. However, the first two implants showed a poor torque during the last rotation, resulting in a lack of primary stability. The position of the implants did not coincide with the digital position as planned in the software.

The explanation of the complication can be referred to a sum of different errors as reported by Valente et al. [14] and summarized in the following.

The different types of errors which could occur during a CT guided implant surgery and prosthetics are as follows.

1. Errors during image acquisition and data processing, on average less than 0.5 mm.
2. Error during surgical template production, typically around 0.1 to 0.2 mm for CAM with stereo-lithography.
3. Error during template positioning and movement of the template during the drilling.
4. Mechanical error caused by the bur-cylinder gap.
5. In template-assisted surgery, the height of the template necessitates very long burs.

In particular, it is evident that the support area of the denture was extremely reduced, and the surgical guide, with 5 holes, has still less contact with the mucosa.

In this case report, the desire to conclude the treatment with an immediate delivery of the prostheses a superficial valuation of the impact to perform three holes so close in the mandible and the subsequent increase of load during mastication due the implant borne prosthesis. Likewise, the presence of 5 splinted implants reduce the elasticity of the mandible during the opening of the mouth. Moreover, the splinted implants might cause an increase of stress in the distal bone [15], exactly in the zone of the hairline fracture. 

**Figure 13:**
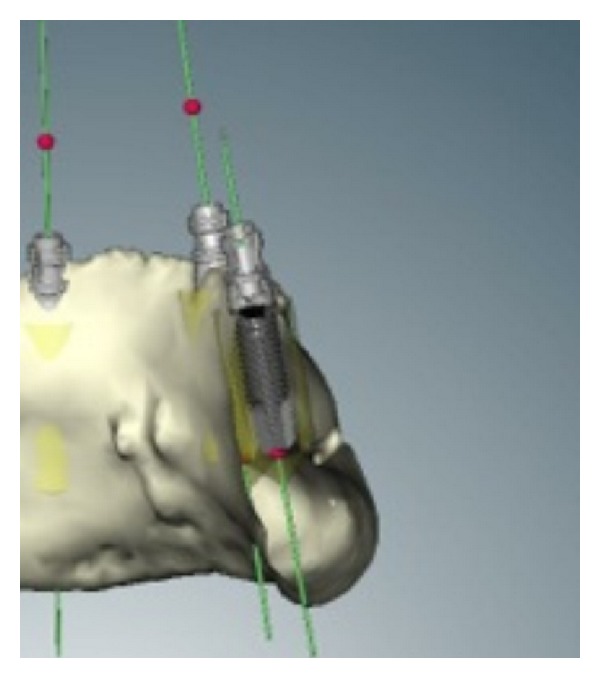
The yellow shade representing the safety area.

The NobelGuide system is a reliable treatment modality, but not without its complications. Strict adherence to the system protocol is only one key to prevent complications. Likewise, it is important during the planning to respect all maximum deviations as the safety margins [16] recommended by the planning software (Figure 13). 

As well as it is important to do the calibration of the scan obtaining the minor deformation of the surgical template. Finally, it is useful a verification of the volumetric congruence of the produced surgical guide and the scan denture [13]. This verification can be attained using a vinyl polyether silicone (VPS) to check the fit of the surgical guide. The thickness of the VPS material indicates space between the guide and the tissue.
